# Age-Related Differences in Arm and Trunk Responses to First and Repeated Exposure to Laterally Induced Imbalances

**DOI:** 10.3390/brainsci10090574

**Published:** 2020-08-20

**Authors:** Ruth Y. Akinlosotu, Nesreen Alissa, John D. Sorkin, George F. Wittenberg, Kelly P. Westlake

**Affiliations:** 1 Department of Physical Therapy and Rehabilitation Science, University of Maryland School of Medicine, Baltimore, MD 21201, USA; Ruakinlosotu@som.umaryland.edu (R.Y.A.); Nesreen.alissa@som.umaryland.edu (N.A.); 2 Baltimore VA Medical Center Geriatrics Research, Education, and Clinical Center, Baltimore, MD 21201, USA; Jsorkin@som.umaryland.edu; 3 Department of Medicine, University of Maryland School of Medicine, Baltimore, MD 21201, USA; 4 VA Maryland HealthCare System, Department of Neurology, University of Maryland School of Medicine, Baltimore, MD 21201, USA; geowitt@pitt.edu; 5 Department of Neurology, University of Pittsburgh School of Medicine, Pittsburgh, PA 15213, USA

**Keywords:** balance, falls, fear of falling, arm movement, postural perturbation

## Abstract

The objective of this study was to examine age-related differences in arm and trunk responses during first and repeated step induced balance perturbations. Young and older adults received 10 trials of unpredictable lateral platform translations. Outcomes included maximum arm and trunk displacement within 1 s of perturbation and at first foot lift off (FFLO), arm and neck muscle activity as recorded using electromyography (EMG), initial step type, balance confidence, and percentage of harness-assisted trials. Compared to young adults, older adults demonstrated greater arm and trunk angular displacements during the first trial, which were present at FFLO and negatively associated with balance confidence. Unlike young adults, recovery steps in older adults were directed towards the fall with a narrowed base of support. Over repeated trials, rapid habituation of first-trial responses of bilateral arm and trunk displacement and EMG amplitude was demonstrated in young adults, but was absent or limited in older adults. Older adults also relied more on harness assistance during balance recovery. Exaggerated arm and trunk responses to sudden lateral balance perturbations in older adults appear to influence step type and balance recovery. Associations of these persistently amplified movements with an increased reliance on harness assistance suggest that training to reduce these deficits could have positive effects in older adults with and without neurological disorders.

## 1. Introduction

Balance perturbations trigger a complex coordination of the arms, legs, and trunk in an effort to restore stability. During slips, trips, or waist pulls, muscle activation of the arms and legs have all been found to occur at comparable latencies, suggesting the presence of a common neural mechanism regardless of perturbation type [1,2]. The tight coupling between these body segments is also evident by the more pronounced responses in the leg and trunk when the arms are constrained compared to when they are free [3]. Although a majority of studies have focused on lower limb stepping responses during whole-body balance perturbations, responses of the arms have also been identified as an important factor in balance recovery [4]. While stepping serves to widen the base of support to increase stability, arm movements can reverse the center of mass excursion, thereby counterbalancing inertial forces in the direction of a fall [5,6].

Older adults demonstrate a natural and robust tendency to incorporate arm movements during attempts to prevent a fall. Using video observations in nursing home settings, this tendency was seen in a majority of falls [7,8,9], although with minimal effectiveness against head impact [8,9]. Even in laboratory-based settings when arm use is restricted either by holding an object or folding them over the chest, older adults are more likely to incorporate the arms into balance responses to externally induced perturbations than young adults [10,11]. Age-related differences in the strategy of these arm responses have also been observed. Young adults generate early arm movements in a direction opposite to trunk displacements, whereas arm movements in older adults are in the same direction as the trunk following unexpected support surface perturbations with feet restrained [12,13]. The responses in older adults are likely an attempt to arrest a fall, but at a potential cost of drastically limiting the ability to restore upright stability [6] and may be a critical factor to the increased risk of falling in this population.

It has been suggested that early arm responses to unexpected imbalances demonstrate a stereotypical startle response upon which a directionally and velocity specific counterbalancing response is incorporated [14,15]. Startle characteristics, such as early and amplified muscle activity and excessive joint angular displacements, are most evident in the first trial and are generally quick to diminish over repeated trials through the general behavioral process known as habituation [14,15]. Fear-potentiated startle responses, on the other hand, have been described as an increase in amplitude of the normal startle response in conditions paired with fearful stimulus [16]. Evidence of increased arm angular displacement and delayed habituation over repeated trials in older compared to young adults suggests the possibility of an underlying conditioned fear of falling with age. These exaggerated responses have been found to negatively correlate with measures of stability at ground impact after a sudden and unexpected vertical drop [17], suggesting a potentially important influence on balance recovery and fall prevention.

Despite evidence of arm use as an integral component of balance reactions and findings that the first perturbation trial represents a unique and possibly fear potentiated response, no studies to date have characterized age-related changes in exaggerated arm movements during first and repeated trials as part of a whole-body balance recovery response. Therefore, the overall objective of this study was to compare arm and trunk responses in relation to protective stepping between young and older adults on first and subsequent lateral perturbation trials. We hypothesized that first-trial responses in older adults would include greater shoulder, elbow, and trunk displacements with reduced habituation compared to young adults and that the extent of arm movement would relate to balance confidence. Secondarily, we expected that the age-related differences in arm and trunk displacement would be evident prior to first foot lift off, resulting in differences in recovery step direction and a need for harness assistance.

## 2. Materials and Methods

### 2.1. Participants

Eleven young adults (7 female, 4 male), with a mean ± standard deviation age of 25.8 ± 3.5 yrs, average mass of 68 ± 11.1 kg, height of 173 ± 9.5 cm, and BMI of 22.8 ± 4.0 kg/m^2^, and 11 older community dwelling adults (8 female, 3 male)—age 70.3 ± 5.4 yrs, mass 75.6 ± 13.8 kg, height 166.5 ± 9.5 cm, and BMI 27.3 ± 4.6 kg/m^2^—participated in this study. All participants reported that they were right-handed except one older and one young adult, who reported being ambidextrous. Individuals with neurological or musculoskeletal conditions affecting activities of daily living (indicated by a score <6/6 on the Katz Index of Independence in Activities in Daily Living) [18] were excluded. It should be noted that this study used a convenience sample of *n* = 11 per group based on previous similar studies [10,17,19,20] and, as a result, was only powered to detect very large effects. This study was reviewed by the University of Maryland Institutional Review Board (Protocol number HP-00089441) and all participants provided consent prior to participating.

### 2.2. Protocol

The ActiveStep Simbex (Simbex, Inc, Lebanon, NH, USA) instrumented treadmill was used to trigger lateral perturbations. Lateral perturbations were used due to the marked vulnerability to falls and fall risk in the lateral direction compared to the anterior/posterior direction in older adults [21,22,23]. Participants assumed a comfortable stance with arms by their sides and looked straight ahead while standing laterally. Instructions were to “do whatever is necessary to restore balance, including taking a step”. To minimize anticipatory postural adjustments, participants were visually monitored to ensure that an upright, relaxed posture with arms hanging freely at the sides was maintained prior to each perturbation trial. An overhead safety harness was worn to prevent actual falls but which allowed participants to freely take a step towards the right or left. A load cell (Noraxon, Scottsdale, AZ, USA) was attached to the harness to determine the amount of harness assistance required during each trial and thus evaluate whether a fall would have occurred using established criteria [24]. ‘Fall’, ‘harness-assist’, or ‘recovery’ trials were defined as ones in which >30%, 5–30%, and <5% body weight was recorded by the load cell, respectively. Each platform translation consisted of a quick 7.67 m/s^2^ acceleration over 90 ms followed by a deceleration of 4.93 m/s^2^ over 140 ms and a displacement of 0.14 m. A total of 10 trials were administered to each participant. Platform translations were all towards the right. We specifically selected a single perturbation direction in order to identify first-trial responses compared to a series of an identical repeated stimuli as has been done by others studying first-trial responses and habituation [17,25,26]. However, to reduce anticipation, participants were told that the perturbation could be towards the right or left and the timing of the perturbation was randomized with an intertrial interval of 30–60s.

### 2.3. Data Collection

Surface electromyographic (EMG) recordings of muscle activation were collected with electrodes placed in accordance with established guidelines (seniam.org) over bilateral sternocleidomastoid (SCM), biceps, and middle deltoid muscles. Muscle onset times were determined using data recorded from a Noraxon TeleMyo wireless system (Noraxon, Scottsdale, AZ, USA) with a sampling frequency of 1500 Hz. The raw surface electromyography (EMG) signals were processed using Visual 3D software (C-motion Inc., Germantown, MD, USA). Signals were bandpass (20–500 Hz) and low-pass filtered (50 Hz) using a digital 4th-order Butterworth filter. Data were then smoothed using the root mean squared (RMS). EMG onset latencies from the time of platform perturbation onset were defined as the time at which the RMS EMG signal exceeded three standard deviations above the mean baseline signal (calculated over the 100 ms prior to platform trigger) and was maintained for 100 ms. EMG amplitude was normalized to the maximum value for each trial within 1 s after perturbation. In addition, we calculated the time to peak EMG amplitude during the first and last trial as the difference between the time of EMG onset and peak amplitude.

Maximum harness-supported loads during each perturbation trial were recorded from a loadcell attached in series with the harness and calculated as the difference between the maximum and minimum points on the loadcell data curve within 1 s after perturbation onset and expressed as a percentage of each participant’s total body mass. Trials were labelled as ‘fall’, ‘harness assist’, and ‘recovery’ using the above-defined criteria.

Movement kinematics were also recorded. The ActiveStep treadmill was surrounded by 6 infra-red motion capture cameras which were driven by Nexus software version 1.8.5 (Vicon Bonita, Vicon Motion Systems, Culver City, CA, USA) at a sampling rate of 100 Hz. At this sampling frequency, the positional accuracy for high speed movements is 2 mm [27]. Reflective markers were placed on 33 anatomical and technical landmarks, including the head (left and right anterior, left and right posterior), neck (7th cervical vertebrae), trunk (sternal notch, left and right mid back), pelvis (sacrum, right and left anterior superior iliac spine), upper arm (bilaterally on the acromion processes, shoulder joint, midpoint of the upper arm (2), forearm (medial and lateral epicondyle, mid-forearm, radial styloid and ulnar processes), and hand (5th metal-carpal, thumb, and index finger). A kinematic model was built using Vicon Nexus software and was further processed using Visual3D (C-Motion, Germantown, MD, USA). Maximum joint angles within 1 s after perturbation onset and joint angles at the time of first foot lift off (FFLO) were determined for bilateral shoulder abduction, elbow flexion, and trunk lateral flexion. Stepping responses during perturbations were video recorded to determine the step type and direction (i.e., widened or similar base of support, BOS, on right or narrowed BOS towards left) and timing of FFLO with respect to perturbation onset using Kinovea software version 0.8.15.

In addition, the Activities-Specific Balance Confidence (ABC) Scale was administered to each participant, which is a 16-item valid and reliable questionnaire measuring self-perceived confidence in performing mobility tasks in and outside the home [28]. ABC scores range from 0 to 100%, with higher scores representing greater balance confidence. There is evidence to suggest that balance confidence influences an individual’s perceived limits of stability, which may in turn influence the response to a quick perturbation [29]. Therefore, we were specifically interested in evaluating the relationship between balance confidence and first trial exaggerated responses. We targeted the shoulder in this analysis due to the consistent occurrence of shoulder abduction as part of a classic startle response compared to more distal arm muscles [30] and the possible influence of this response on balance recovery [14].

### 2.4. Statistical Analysis

All statistical analysis was conducted using SPSS software version 26.0 (IBM, Armonk, NY, USA). A q–q plot and Mauchly’s sphericity test were first used to test the assumption of normality and sphericity of the data, respectively. To detect differences in first and last trial joint angles, EMG onset latencies, and EMG time to peak amplitude between young and older groups, two-way repeated-measures ANOVAs were used with group (young vs. older) as the between-subject factor and trial number as the within-subject factor (trial 1 vs. 10). To determine group differences in the rate of habituation on max joint angles and EMG amplitude (each trial expressed as percentage of 1st trial), two-way repeated-measures ANOVAs were used with group (young vs. older) as the between-subject factor and trial number (trial 1 vs. 2–10) as the within-subject factor. Bonferroni multiple comparisons tests were used for the ANOVAs in order to compute a multiplicity adjusted *p*-value for each pairwise comparison in cases of significant main and interaction effects. To assess the presence of age-related differences in anticipatory muscle activity, an independent *T*-test was used to determine group differences in EMG activity during the 100 ms prior to perturbation onset. A Pearson chi-square test was used to analyze differences in step type between young and older adults for trials 1 and 10. A Spearman Rank Order Correlation was run to determine the relationship between ABC scores and maximum shoulder joint angles in the older participants. The level of significance was established at alpha = 0.05 for all analyses.

## 3. Results

### 3.1. First and Last Trial Age-Related Differences

**Figure 1:**
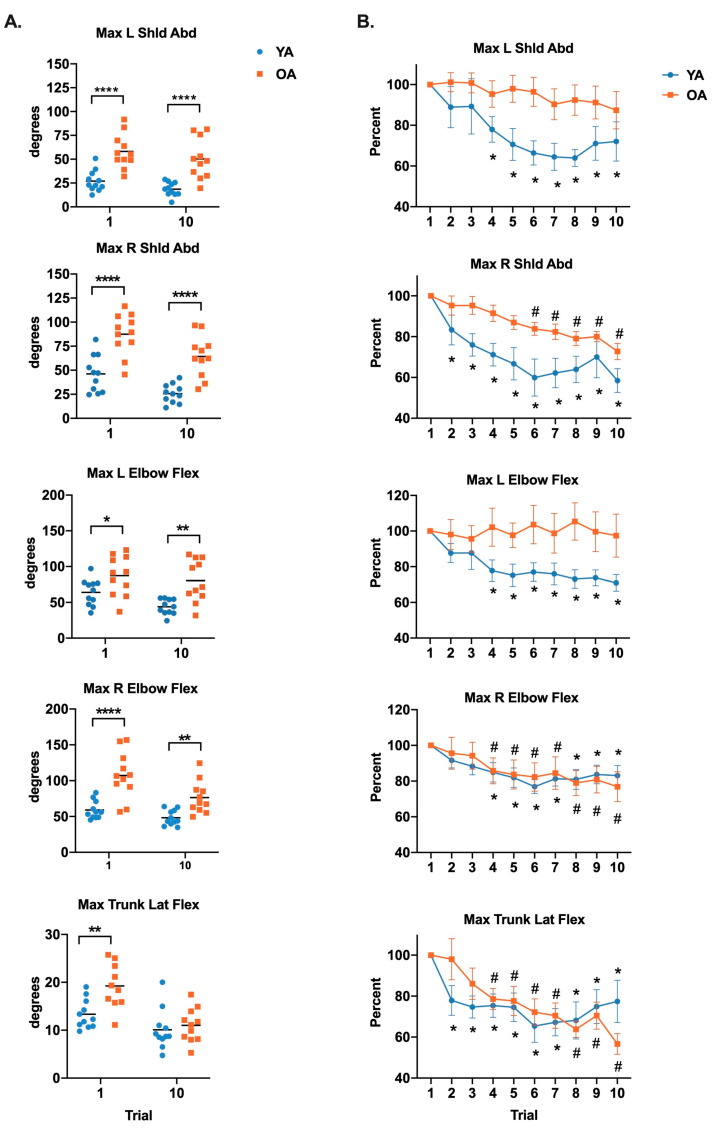
Age-related differences in displacements of shoulder abduction, elbow flexion, and left lateral trunk flexion during trials 1 and 10 (**A**) and within-group habituation over 10 successive trials (**B**). Values in scatterplots in the left column are expressed as the mean (horizontal lines) and individual data points. Values in the right column are expressed as the mean +/− standard error. Left column, corrected *p*-values depict differences between young and older adults. * *p* < 0.05, ** *p* < 0.01, and **** *p* < 0.0001. Right column corrected *p*-values depict within-group differences between trial 1 and each of the subsequent trials. * *p* < 0.05 in young group and # *p* < 0.05 in older group.

Figure 1A illustrates maximum joint angles of the shoulder, elbow, and trunk at trials 1 and 10 for young and older adults. A significant main effect of age was found for all joints, with an interaction between group and trial only for trunk angle. More specifically, older adults demonstrated greater joint angles than young adults at both trials 1 and 10 for shoulder abduction (left *F*_(1,20)_ = 28.80, *p* < 0.0001; right *F*_(1,20)_ = 28.90, *p* < 0.0001), elbow flexion (left *F*_(1,20)_ = 13.85, *p* = 0.001; right *F*_(1,20)_ = 28.52, *p* < 0.0001) and left trunk lateral flexion (*F*_(1,20)_ = 6.34, *p* = 0.02). Post hoc analyses of the interaction for left lateral trunk flexion (*F*_(1,20)_ = 7.16, *p* = 0.01) indicated greater lateral flexion in older compared to young adults in trial 1 (*p* = 0.002), but not during trial 10 (*p* > 1.0).

**Figure 2:**
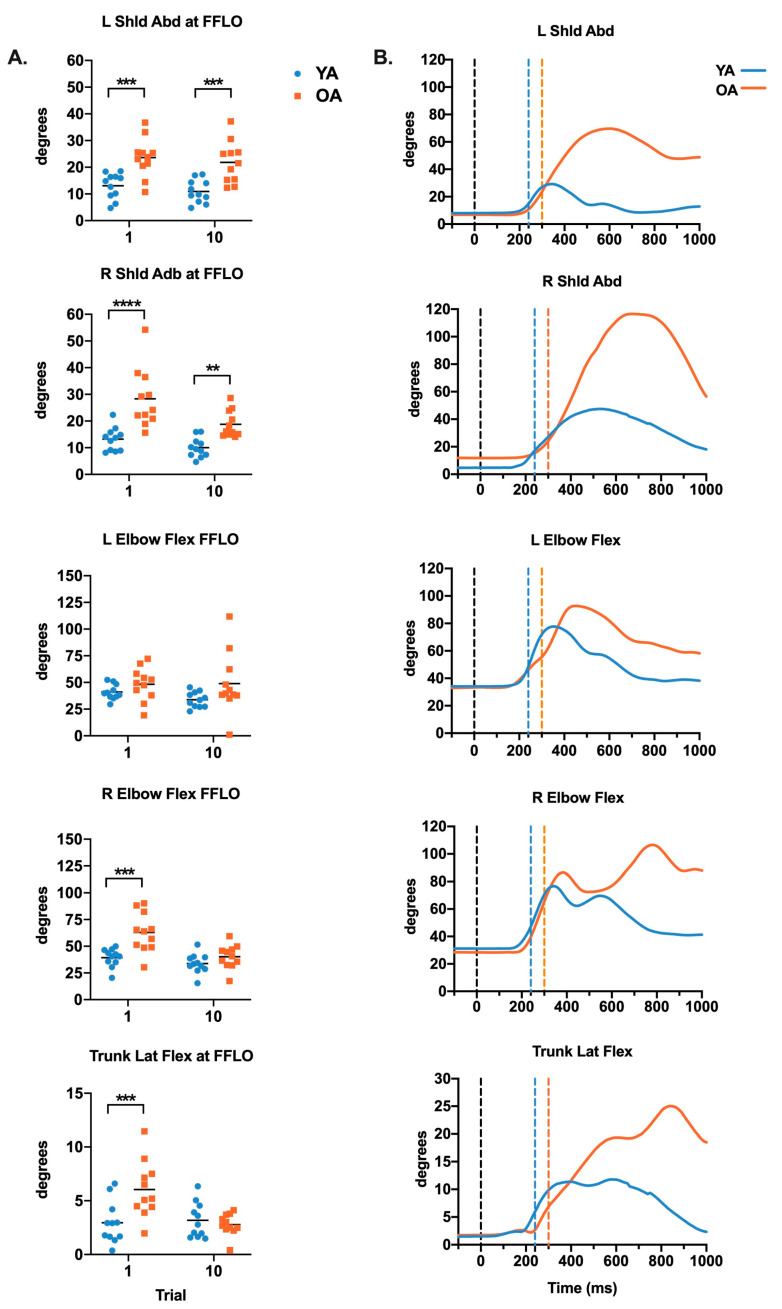
(**A**) Age-related differences in displacement of shoulder abduction, elbow flexion, and left lateral trunk flexion at first foot lift off (FFLO) in trial 1 and 10. Values in scatterplots in the left column are expressed as the mean (horizontal lines) and individual data points. Corrected *p*-values depict differences between young and older adults, ** *p* < 0.01, *** *p* < 0.001, and **** *p* < 0.0001. (**B**) Joint angle vs. time curves for a representative young and older adult. The black vertical line depicts the onset time of platform perturbation and the time of FFLO is labeled using a blue vertical line for young adults and an orange vertical line for older adults.

Figure 2 illustrates joint angles at the time of FFLO. Again, significant main effects of age were identified for bilateral shoulder abduction with greater joint angles in older compared to younger adults were found for shoulder abduction (left *F*_(1,20)_ = 21.39, *p* = 0.0002; right *F*_(1,20)_ = 25.15, *p* < 0.0001). An interaction between group and trial for right elbow flexion (*F*_(1,20)_ = 10.17, *p* = 0.005) and lateral trunk flexion (*F*_(1,20)_ = 12.16, *p* = 0.002) demonstrated greater angles in older compared to young adults during trial 1 (right elbow, *p* = 0.0001; lateral trunk, *p* = 0.0009), but not trial 10 (right elbow, *p* = 0.49; lateral trunk, *p* > 1.0;). No significant group differences were identified for left elbow flexion at FFLO (*F*_(1,20)_ = 2.82, *p* = 0.11).

Details regarding onset latencies are outlined in Table 1. There was no main effect of age or trial nor were there any interaction effects in mean EMG onset latencies when comparing trial 1 and 10 in either group for SCM (left *F*_(1,20)_ = 0.071, *p* = 0.79; right *F*_(1,20)_ = 0.275, *p* = 0.61), mid deltoid (left *F*_(1,20)_ = 0.3, *p* = 0.59; right *F*_(1,20)_ = 0.884, *p* = 0.36), or biceps (left *F*_(1,20)_ = 0.17, *p* = 0.68; right *F*_(1,20)_ = 0.895, *p* = 0.36) between the older and younger adults. However, a main effect of group was identified for FFLO onset latencies, which were delayed in older compared to young adults (*F*_(1,20)_ = 5.527, *p* = 0.03).

Table 2 shows the mean and standard error of the time to peak in older and younger adults for trial 1 and 10. An interaction effect of the log-transformed data between age and trial was noted for right middle deltoid (*F_(_*_1,20)_ = 5.13, *p* = 0.04), while a main effect of age was noted for left SCM (*F*_(1,20)_ = 7.25, *p* = 0.01), left middle deltoid (*F*_(1,20)_ = 5.81, *p* = 0.03), and biceps (left *F*_(1,20)_ = 6.87, *p* = 0.02; right *F*_(1,20)_ = 7.74, *p* = 0.01), but not for right SCM (*F*_(1,20)_ = 0.20, *p* = 0.66).

### 3.2. Habituation

Figure 1B illustrates habituation effects as the trial in which maximum joint angle (normalized to the angle in the first trial) decreased to a value that was significantly different from the first trial. Interaction effects were identified for left shoulder abduction (*F*_(9,180)_ = 1.994, *p* = 0.04), left elbow flexion (*F*_(9,180)_ = 3.255, *p* = 0.001), and left lateral trunk flexion (*F*_(9,180)_ = 2.30, *p =* 0.02). Post hoc analyses revealed that habituation in the young adults occurred at trial 4 for left shoulder abduction (*p* = 0.01) and left elbow flexion (*p* = 0.001) but did not occur within the 10 trials in older adults (left shoulder abduction *p* = 0.55 at trial 10; left elbow flexion *p* > 1.0 at trial 10). For left lateral trunk flexion, habituation occurred by trial 2 in young adults (*p* = 0.01) and in trial 4 in older adults (*p* = 0.03).

**Figure 3:**
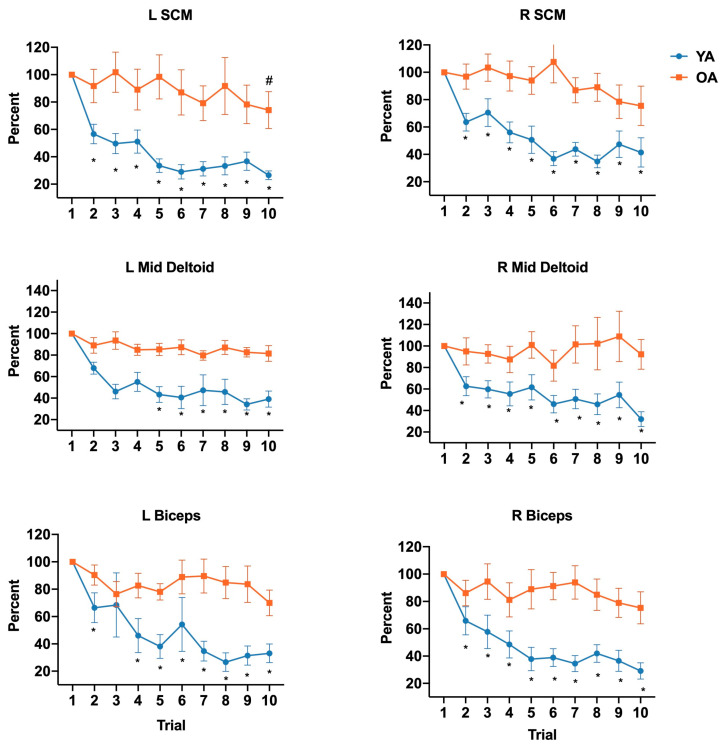
Age-related differences in habituation rates of muscle amplitude (right and left middle deltoid, biceps, SCM) over 10 successive trials. Values are expressed as the mean ± standard error. The asterisk (*) and hashtag (#) depict significant differences between trial 1 and subsequent trials for young and older adults, respectively.

Differences in EMG amplitude habituation rates in older and younger adults are displayed in Figure 3. With the exception of left mid deltoid, which had a significant main effect of trial on habituation rate (*F*_(9,180)_ = 3.36, *p* = 0.0008), there were significant interactions between group and trial for all muscles, SCM (left *F_(_*_9,180)_ = 3.99, *p =* 0.0001; right *F*_(9,180)_ = 3.19, *p* = 0.001), biceps (left *F*_(9,180)_ = 3.16, *p* = 0.001; right *F*_(9,180)_ = 3.24, *p* = 0.001), and right mid deltoid (*F*_(9,180)_ = 1.95, *p* = 0.05). Post hoc analyses revealed that habituation in young adults occurred as early as trial 2 for nearly all muscles, SCM (left, *p* = 0.0001; right, *p* = 0.004); right mid deltoid *p*= 0.03; biceps (left, *p* = 0.03; right, *p* = 0.002) and trial 5 in mid deltoid (*p* = 0.004). In contrast, in the older adults, none of the muscles showed habituation within the 10 trials except left SCM at trial 10 (*p* = 0.04).

In order to determine whether the age-related differences in habituation were due to the young adults employing anticipatory postural control mechanisms prior to perturbation onset, we assessed age-related differences in baseline EMG activity during the 100 ms prior to perturbation. Results of this analysis revealed that there were no significant group differences for any of the muscles tested [left SCM *p* = 0.67; right SCM *p* = 0.4; left mid deltoid *p* = 0.11; right mid deltoid *p* = 0.16; left biceps *p* = 0.34; right biceps *p* = 0.25). The average EMG amplitude +/− SEM within 100 ms prior to perturbation was as follows (young, older): left SCM = 5.73 mv ± 1.65mv, 4.97 ± 0.56 mv; right SCM = 4.13 ± 0.46 mv, 4.71 ± 0.49 mv; left mid deltoid = 3.55 ± 0.34 mv, 6.72 ± 1.87 mv; right mid deltoid = 3.66 ± 0.41 mv, 4.90 ± 0.74 mv; left biceps = 3.08 ± 0.37 mv, 3.74 ± 0.0.57 mv; right biceps = 2.94 ± 0.33 mv, 3.90 ± 0.74 mv.

### 3.3. Step Types

**Figure 4:**
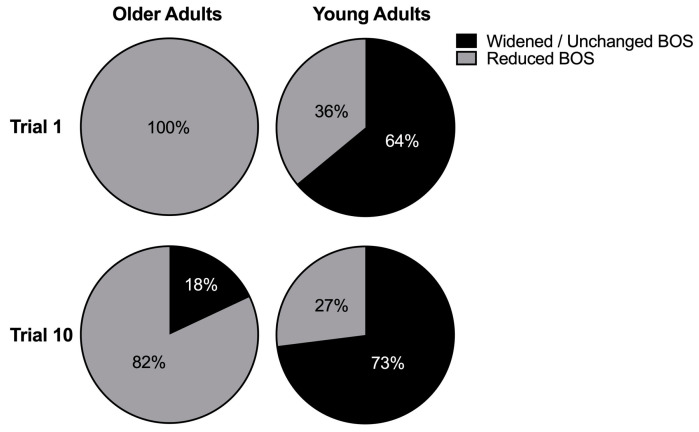
Percent of narrowed and widened/unchanged base of support following a right step in older and young adults in trial 1 and 10. Narrowed based of support was towards the left and into the direction of the fall and widened or unchanged base of support was towards the right in a counterbalancing direction away from the fall.

Results of step types taken by the older and younger adults are depicted in Figure 4. We classified first step types as ones that resulted in an increased or unchanged BOS (i.e., step on the right to either widen BOS or return to the original BOS) or reduced BOS (i.e., medial, cross-back, and cross front step types to the left). A chi-square analysis of all the step types across the ten trials revealed significant differences between older and younger adults (χ^2^ = 157.7, *p* = 0.001). Post hoc testing revealed a significant difference in step types between the older and younger adults in both trial 1 (*p* < 0.001) and trial 10 (*p* < 0.001). The majority of first steps taken by older adults resulted in a narrowed BOS with a right leg step towards left, while most first steps in the young adults resulted in an increased or unchanged BOS with a right leg step on right.

### 3.4. Harness Assistance and Balance Confidence

With the exception of one younger adult, older and younger adults were able to restore balance without an in-task incidence of a *fall* (defined as >30% BW support). However, in older participants, 91% required harness assist (i.e., 5%–30% BW support) during trial 1 and 64% required harness assist by trial 10. Among the younger adults, only 27% harness-assisted trials were recorded in the 1st trial, which reduced to 18.2% by the 10th trial. Moreover, among the older adults, a significant relationship was found between ABC scores and right (*r* = −0.639, *p* < 0.05), but not left, shoulder abduction during the first trial.

## 4. Discussion

Overall, results of this study support our hypotheses by demonstrating age-related differences in exaggerated first-trial arm and trunk responses to sudden lateral balance perturbations, which appear to influence balance recovery. Moreover, although age-related differences in lateral trunk displacement were minimized by trial 10, greater joint displacement of both arms were still evident by the 10th trial in older compared to younger adults. In addition, comparisons with the first trial revealed rapid habituation in bilateral arms and trunk in the young adults, but asymmetrical habituation of only the right arm and lateral trunk responses in older adults.

Maximum joint displacements following first perturbation exposure were on average, 35 degrees greater for shoulder abduction and elbow flexion in older compared to young adults, with greater differences on the right. The net effect of the exaggerated arm movement, in combination with greater left lateral trunk flexion in the direction of the fall and delayed first foot lift off, is center of mass displacement towards the left, generally resulting in a need for harness assistance during balance recovery. All of the older adults took a step with the unloaded right foot towards the left that narrowed the base of support compared to only 36% of younger adults. These steps included cross- over steps, which have been shown to be associated with a higher rate of limb collisions and fall risk [31]. In contrast, young adults tended to counterbalance the leftward-directed COM displacement by extending the right arm towards the right and likely actively engaging trunk musculature to effectively counterbalance the leftward-directed COM displacement. Similar age-related differences in balance recovery strategies have been identified during feet restrained conditions in which initial arm and trunk movements are in the direction of the fall in older adults, possibly to protect against the fall, and in a counterbalancing direction in young adults [12].

The tendency for exaggerated arm movements may be a response to either a perceived or real instability. Support for a perceived instability includes the negative relationship identified between balance confidence and shoulder displacement. Although the sample size for this correlation was small, the finding supports previous reports of a direct link between fear of falling and changes in gait and perceived limits of stability under conditions of postural threat [32,33,34,35]. In addition, the pattern of responses, with shoulder abduction and elbow flexion, share similar characteristics with the classic startle response [36]. However, the lack of a typical rostrocaudal progression in EMG onset latencies suggests a task dependency that was not due to differences in background EMG activity. Others have reported task dependent startle responses to loud auditory sounds during standing compared to sitting and at different phases of gait [37,38]. Thus, these results may support the functional relevance of a startle response, albeit more pronounced on the older adults, which is meant to protect against falls and/or restore stability. On the other hand, age-related differences in arm and trunk responses may reflect real instability as the consequences of age-related decrements in musculoskeletal capacity of the trunk and hips, sensory function of the lower limbs and trunk, and sensory integration of vestibular and proprioceptive inputs [39,40,41]. The delays in FFLO, which have also been previously reported [42], support the presence of musculoskeletal limitations, including the possibility of weak and decreased rate of activation of the hip abductors/adductors and internal oblique/transversus abdominus [42,43,44] or reduced transmission or integration of sensory information regarding the characteristics and direction of the balance perturbation [39,40]. It is also possible that trunk stiffness due to degenerative changes and increased muscle stiffness [12], active muscular co-contractions (i.e., due to fear of falling) [45], or a “stiffening” strategy as described in some patients with excessive startle reactions may contributed to the increased left lateral trunk flexion in older adults during the early trials following the initially passive perturbation-driven displacement. However, it is important to note that by trial 10, lateral trunk displacement was similar to young adults, suggesting that the cause of initial age-related differences was readily reversable. Thus, future research into the underlying mechanisms for the exaggerated arm strategy, particularly in relation to the trunk and stepping response are needed.

The rate at which habituation of exaggerated first-trial responses occurred also demonstrated age-related differences. In general, young adults scaled down arm and trunk movement and EMG amplitude to match perturbation characteristics within 2 to 5 trials, whereas only the trunk and right arm movement was reduced in older adults, occurring by trials 4 to 6, respectively, with no change in EMG amplitude over the repeated trials. Our findings of age-related differences in habituation of EMG amplitude are largely similar to Sanders et al. (2019), who reported rapid habituation in SCM, mid deltoid, and biceps in younger adults and no habituation in the first 10 trials of older adults except in mid deltoid during a sudden drop perturbation [17]. A rapid habituation rate in young adults has also been previously identified and underscores the superfluous nature of movements observed during responses to the first unexpected trial [14,17,46].

In the older adults, we can speculate several reasons for the lack of or delayed habituation in the joint angles and EMG amplitude compared to young adults. The first being, as noted above, the persistent sense of perceived or real instability, which was much more pronounced and sustained in the arms of older adults. The second being an impaired predictive capacity, which is essential for the preparation and execution of efficient balances response upon repeated trials with precisely the same characteristics. Third, findings of increased time to peak EMG amplitude during both the first and last trial in older compared to young adults in nearly all muscles suggests the possibility of impaired rates of arm muscle activation. Only right middle deltoid demonstrated a faster time to peak by trial 10, which is in line with the habituation found in right shoulder kinematics. Although the EMG amplitude may be similar between first and subsequent trials, the time to achieve to peak activation was reduced. Similar age-related decreases in the rate of muscular activation have been found at the hip during protective stepping responses [47]. Forth, and also as noted above, it has been suggested that a startle reflex is superimposed upon a directionally specific balance response during the first trial [15,48] and then gets extinguished during subsequent trials, leaving only the postural response [49]. The persistently exaggerated displacement of the arms of older adults in the current study may therefore reflect a fear potentiated startle response. This abnormal response has been described as one that is conditioned to an encounter with fearful stimuli and elicits a larger response than a neutral startle stimulus and is sustained over multiple trials [16]. The asymmetrical joint displacement during the first trial and presence of habituation in the right, but not the left arm of older adults lends support to this theory. The rightward-directed perturbation induced a leftward-directed fall such that the lack of habituation in the left arm may reflect the ongoing perceived state of instability and the resulting tendency for this arm to move into a protective position against impact. In contrast, the right arm response demonstrated an initially exaggerated response that later diminished as it was not as critical to a leftward fall direction. It should also be noted that habituation is a simple form of motor learning, with both feed-forward and feed-back control mechanisms. Although our results show no evidence of anticipatory EMG activity and the onset latencies in EMG activity were not different between trials 1 and 10 in either group, we were unable to monitor pre-loading of the limb or activation of the trunk muscles prior to perturbation. As a result, we cannot rule out the possibility that the younger adults may have been better able to use predictive feed-forward control mechanisms than older adults, which could have led to earlier habituation.

Although the first-trial responses display real-world validity for understanding falls, the importance of understanding the process of habituation has implications for determining intervention potential and the development of treatment protocols. For example, despite the lack of habituation in the left arm, findings of relatively rapid trunk habituation in older adults suggest an important capacity to engage core musculature to minimize trunk displacement in the direction of the fall. In addition, the demonstrated ability for older adults to increase the rate of muscle activation of the right arm points to the possibility of training faster activation rates in the arms and trunk for effective counterbalancing. Together, these strategies may represent an important focus for the development of future reactive balance training programs. Nevertheless, with the first recovery step primarily serving to narrow the base of support and a greater number of trials with ‘harness assistance’ in older adults across all 10 trials, consideration should also be given to the influence of arm and trunk responses with respect to protective stepping as a future training target.

Among the study limitations, it should be noted that perturbations were induced with the same characteristics (e.g., distance, velocity, acceleration, and direction) for all participants. An alternative would have been to determine individual balance perturbation threshold level at which point a participant naturally feels the need to step. However, in so doing, the first-trial responses would not have been captured. Based on our prior research, we selected the lowest perturbation magnitude known to consistently induce protective responses in both older and younger adults. Indeed, all participants in the current study required a balance recovery step following the sudden perturbation.

## 5. Conclusions

In summary, findings of exaggerated first-trial arm and trunk responses to sudden lateral balance perturbations in older adults appear to negatively influence balance recovery. The persistently amplified movements and delayed stepping resulted in an increased reliance on harness assistance and appear to involve factors that may be associated with a lack of balance confidence. Our ongoing research seeks to further elucidate the mechanisms underlying impaired first-trial responses of the arms and trunk during reactive stepping perturbations in order to develop targeted interventions and ultimately reduce falls in older adults with and without neurological disorders.

## Figures and Tables

**Table 1:** Mean ± standard error of onset latencies for older and young adult (ms).

Muscle	Older Adults		Younger Adults		*p*-Value
	Trial 1	Trial 1 0	Trial 1	Trial 10	
Left SCM	140.9 ± 12.89	122.8 ± 9.00	127.8 ± 15.18	128.4 ± 12.69	0.79
Right SCM	144.4 ± 14.43	141.9 ± 8.57	124.3 ± 11.53	147.8 ± 12.80	0.61
Left Mid Deltoid	139.6 ± 13.06	131.0 ± 6.74	125.8 ± 10.58	132.5 ± 10.61	0.59
Right Mid Deltoid	142.7 ± 12.37	125.3 ± 8.37	120.6 ± 10.94	125.5 ± 10.37	0.36
Left Biceps	149.6 ± 12.61	129.3 ± 9.42	126.9 ± 11.12	140.9 ± 13.08	0.68
Right Biceps	147.7 ± 13.89	144.7 ± 8.43	128.6 ± 10.95	140.0 ± 11.00	0.36
FFLO Onset Latency	263.6 ± 19.55	226.4 ± 9.17	216.4 ± 8.45	214.5 ± 9.28	0.03 *

SCM = sternocleidomastoid; * significant main effect of group.

**Table 2:** Mean and standard error of time to peak EMG amplitude (ms).

Muscle	Older Adults		Younger Adults		*p*-Value
	Trial 1	Trial 10	Trial 1	Trial 10	
Left SCM	272.9 ± 21.27	167.6 ± 1.16	127.8 ± 1.10	135.6 ± 1.11	0.01 *
Right SCM	161.1 ± 1.24	129.9 ± 1.06	148.2 ± 1.15	122.0 ± 3.59	0.66
Left Mid Deltoid	199.5 ± 8.31	126.0 ± 2.05	122.9 ± 1.06	117.0 ± 1.11	0.03 *
Right Mid Deltoid	307.3 ± 1.25	137.9 ± 1.19	128.3 ± 1.06	137.9 ± 1.13	0.04 †
Left Biceps	221.9 ± 1.22	151.9 ± 1.07	124.8 ± 1.08	136.6 ± 1.13	0.02 *
Right Biceps	319.2 ± 1.25	124.8 ± 1.08	122.7 ± 1.05	147.9 ± 1.19	0.01 *

SCM = sternocleidomastoid; * significant main effect of age; † significant interaction effect between trial and age.
